# Ten new species of the spider genus *Sinoderces* Li & Li, 2017 from China, Laos and Thailand (Araneae, Psilodercidae)

**DOI:** 10.3897/zookeys.886.39212

**Published:** 2019-11-05

**Authors:** Zilong Bai, Fengyuan Li, Shuqiang Li

**Affiliations:** 1 Institute of Zoology, Chinese Academy of Sciences, Beijing 100101, China

**Keywords:** Biodiversity, endemism, ochyroceratids, taxonomy, tropical spiders

## Abstract

Ten new species of the spider family Psilodercidae Machado, 1951 are described from tropical East Asia, including five species found in China: *Sinoderces luohanensis* Li & Li, **sp. nov.** (♂♀), *S. xueae* Li & Li, **sp. nov.** (♂♀), *S. taichi* Li & Li, **sp. nov.** (♂♀), *S. wenshanensis* Li & Li, **sp. nov.** (♂♀), *S. aiensis* Li & Li, **sp. nov.** (♂♀); three are from Laos: *S. khanensis* Li & Li, **sp. nov.** (♂♀), *S. phathaoensis* Li & Li, **sp. nov.** (♂♀), *S. kieoensis* Li & Li, **sp. nov.** (♂); and the rest are from Thailand: *S. saraburiensis* Li & Li, **sp. nov.** (♂), *S. dewaroopensis* Li & Li, **sp. nov.** (♂♀). Types of all new species are deposited in the Institute of Zoology, Chinese Academy of Sciences in Beijing, China.

## Introduction

A great diversity of spiders of the family Psilodercidae Machado, 1951 has evolved throughout the zoogeographic regions of Indo-Burma, Sundaland, the Philippines and Wallacea. To date, 141 psilodercid species have been recorded from the various countries collectively known as “Tropical East Asia” (Li and Quan 2017; World Spider Catalog 2019). They are small, fragile, web-spinning spiders that inhabit dark, damp places, such as leaf litter, tree buttresses and caves. Previously classified as a subfamily within Ochyroceratidae Fage, 1912, Wunderlich (2004) opined that they deserved a family rank as they share several characters that distinguish them from other ochyrocertids (*sensu*Ochyroceratinae): they have book-lungs; their posterior tracheal opening is closer to the spinnerets than other ochyroceratids; they have only 0–3 cheliceral promarginal teeth (as opposed to 6–7 in other ochyroceratids); unlike other ochyroceratids, their labium is not incised; and the position of the bulb is usually at the end of the cymbium, and not near the middle as in other ochyroceratids. The group was subsequently elevated to its own family (Wunderlich 2008).

The genus *Sinoderces* Li & Li, 2017 is currently included in the family Psilodercidae. Only two *Sinoderces* species have been documented so far, namely *S. exilis* (Wang & Li, 2013) and *S. nawanensis* Li & Li, 2017, both from China. In the present paper, we describe ten new species of *Sinoderces* from China, Laos and Thailand.

## Material and methods

All specimens were collected in China, Thailand and Laos (Fig. 22), and preserved in 95% ethanol. Types of all new species are deposited in the Institute of Zoology, Chinese Academy of Sciences in Beijing, China. Specimens were examined and measured using a Leica M205 C stereomicroscope. Morphological details were studied with an Olympus BX41 compound microscope. Photos were taken with an Olympus C7070 wide zoom digital camera (7.1 megapixels) mounted on an Olympus SZX12 stereomicroscope. The images were montaged using Helicon Focus 6.7.1 image stacking software. The map was generated using ArcView GIS 10.2. All measurements are in millimeters (mm). Leg measurements are shown as total length (femur, patella, tibia, metatarsus, and tarsus). Leg segments were measured from the retrolateral side. Carapace length was measured from the anterior eye row to the carapace posterior margin. Terminology follows that of Deeleman-Reinhold (1995), Tong and Li (2007), and Li et al. (2014).

## Taxonomy

### Family Psilodercidae Machado, 1951

#### Genus Sinoderces

Taxon classificationAnimaliaAraneaePsilodercidae

Li & Li, 2017

92BD6536-5C5C-58F1-9DC1-DE927A9C3DD6

##### Type species.

*Sinoderces nawanensis* Li & Li, 2017

##### Diagnosis.

The genus is distinguished (together with *Thaiderces* Li & Li, 2017) from all the other genera in Psilodercidae by the absence of an apical protrusion on the male cymbium. It is distinguished from the genus *Thaiderces* by the presence of a single tooth on the cheliceral retromargin and the long embolus of the palpal bulb (Liu et al. 2017).

Cheliceral promargin with lamina, retromargin with a tooth or denticle; palpal bulb with long embolus; conductor present or absent-if present, then embolus and conductor separated basally; female with two pairs of elongate, curved spermathecae (Liu et al. 2017).

#### Sinoderces khanensis

Taxon classificationAnimaliaAraneaePsilodercidae

Li & Li sp. nov.

B0556279-DDBE-5EA4-A314-D4B37DD4E39A

http://zoobank.org/697B62E8-0DFC-49AA-8DD8-A21FE6F35692

**Figure 1.**
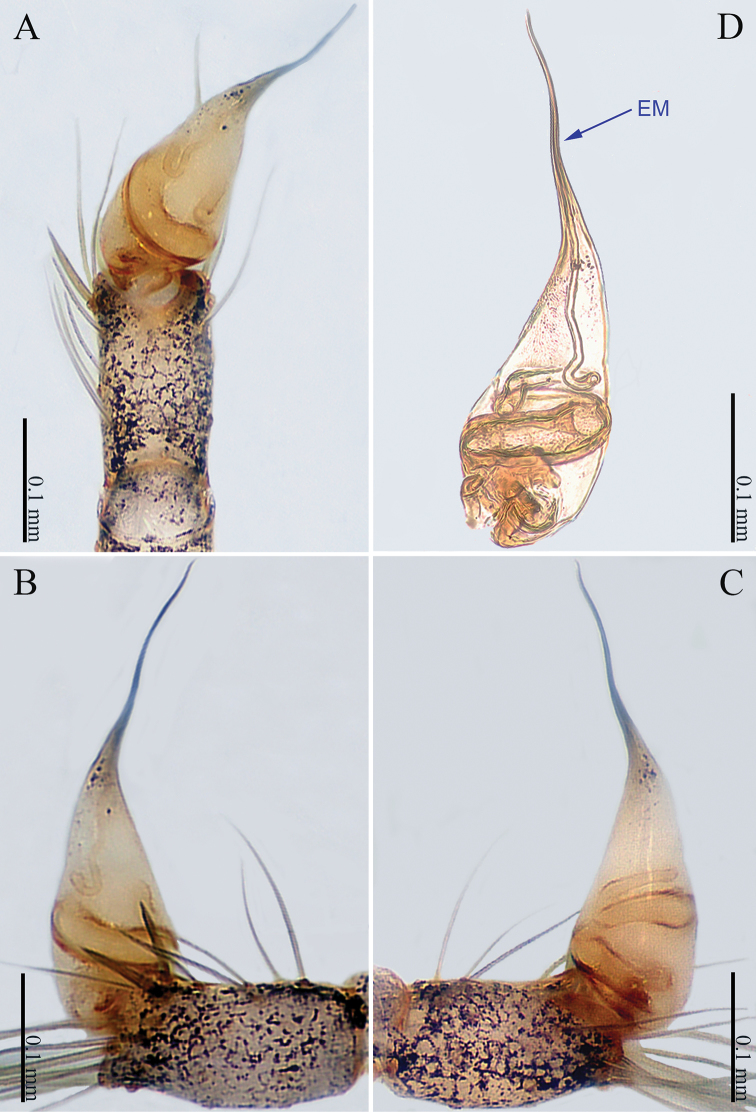
*Sinoderces khanensis* Li & Li, sp. nov., male holotype **A** left palp, ventral view **B** left palp, prolateral view **C** left palp, retrolateral view **D** right palpal bulb, prolateral view. Abbreviation: EM embolus.

**Figure 2.**
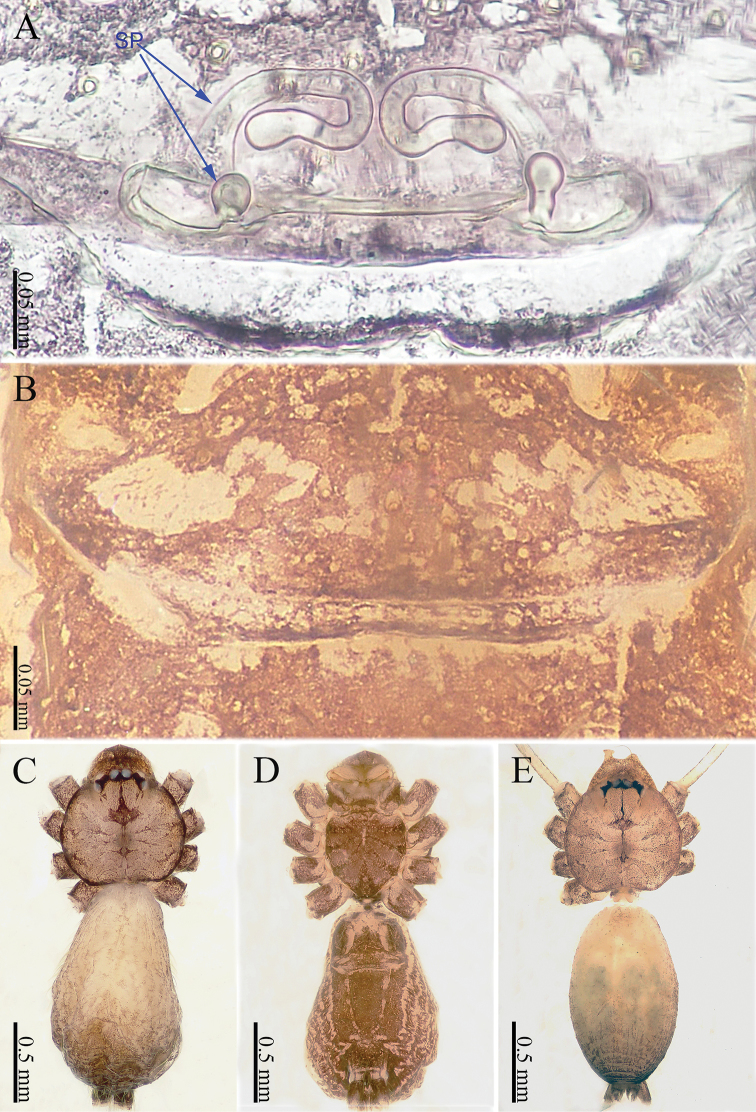
*Sinoderces khanensis* Li & Li, sp. nov., male holotype and female paratype **A** internal genitalia, dorsal view **B** female epigastric furrow, ventral view **C** female habitus, dorsal view **D** female habitus, ventral view **E** male habitus, dorsal view. Abbreviation: SP spermathecae.

Figs 1 , 2 , 21 , 22

##### Types.

***Holotype***: ♂, Khan Cave, 5.35 km west of Viengkieo Village, Vang Vieng District, Vientiane Province, Laos, 18°55.592'N, 102°23.718'E, 270 m, 01.XI.2012, Yao Z. leg. ***Paratypes***: 1♂2♀, same data as holotype.

##### Etymology.

The specific name refers to the cave where type material was collected; adjective.

##### Diagnosis.

Male *Sinoderces khanensis* sp. nov. can be recognized by the overall slender configuration of the palp, with two spines on the cymbium (Fig. 1B). The male clypeus has two apophyses (Fig. 2E). In the female, the spermathecae are extraordinarily elongated and shaped like the front of headband worn by the Chinese mythological Monkey King, Sun Wukong-two blunt ends that meet and curl upward (Fig. 2A).

##### Description.

**Male** (holotype). Total length 2.33; carapace 1.02 long, 0.75 wide; abdomen 1.28 long, 0.70 wide. Clypeus light brown, with two apophyses. Carapace round, light yellow, with brown lateral margins and a wide median brown band, which is noticeably broader in the mid-section (Fig. 2E). Cheliceral promargin with one tooth, connected to a lamina, retromargin with a small tooth (Fig. 21A). Labium light brown. Sternum brown. Legs brownish yellow, with dark brown joints. Leg measurements: I missing, II 7.82 (2.20, 0.20, 2.33, 2.17, 0.92), III 5.54 (1.60, 0.20, 1.80, 1.52, 0.42), IV 8.52 (2.56, 0.20, 2.64, 2.28, 0.84). Abdomen elongated, with small black stripes, the color tone darkens progressively from front to back (Fig. 2E). Spinnerets black.

***Male palp*** (Fig. 1A–D): The overall color is pale yellow. Bulb light yellow, conical. Needle-like embolus arising distally from bulb, straight; no conductor. Tibia with numerous long setae and two conspicuous spines. Long femur with sparse hairs.

**Female** (one of the paratypes). Size and color similar to male (Fig. 2C, D). Total length 1.92; carapace 0.63 long, 0.72 wide; abdomen 1.29 long, 0.76 wide. Clypeus dark brown. Carapace round, darker than that of the male. Labium and sternum dark brown, a white spot in the middle of sternum. Legs brownish yellow, with darkish brown joints. Leg measurements: I missing, II 6.06 (1.56, 0.19, 1.94, 1.56, 0.81), III 4.71 (1.25, 0.19, 1.38, 1.20, 0.69), IV 7.14 (2.03, 0.20, 2.19, 1.84, 0.88). Abdomen elongated, dorsum color tone darkens progressively towards the posterior end; and ventrum with numerous white spots and other markings. Spinnerets dark brown.

***Epigyne*** (Fig. 2A, B): Shaped like a mouth with a thick lip (Fig. 2B). It has two pairs of spermathecae: a pair of small and spherical spermathecae connected to a pair of highly elongated and looped spermathecae. The spermathecae conjure an image of the headband worn by the Chinese mythological Monkey King with two blunt ends that meet and curl upward (Fig. 2A).

##### Distribution.

Known only from the type locality (Fig. 22).

##### Natural history.

Collected at a cave’s entrance at an elevation of 270 m.

#### Sinoderces luohanensis

Taxon classificationAnimaliaAraneaePsilodercidae

Li & Li sp. nov.

799B35DB-C8C5-52A3-A47C-51C23E7E54DC

http://zoobank.org/1E9870D7-4BBD-4DE1-81EA-5E43F9672D05

**Figure 3.**
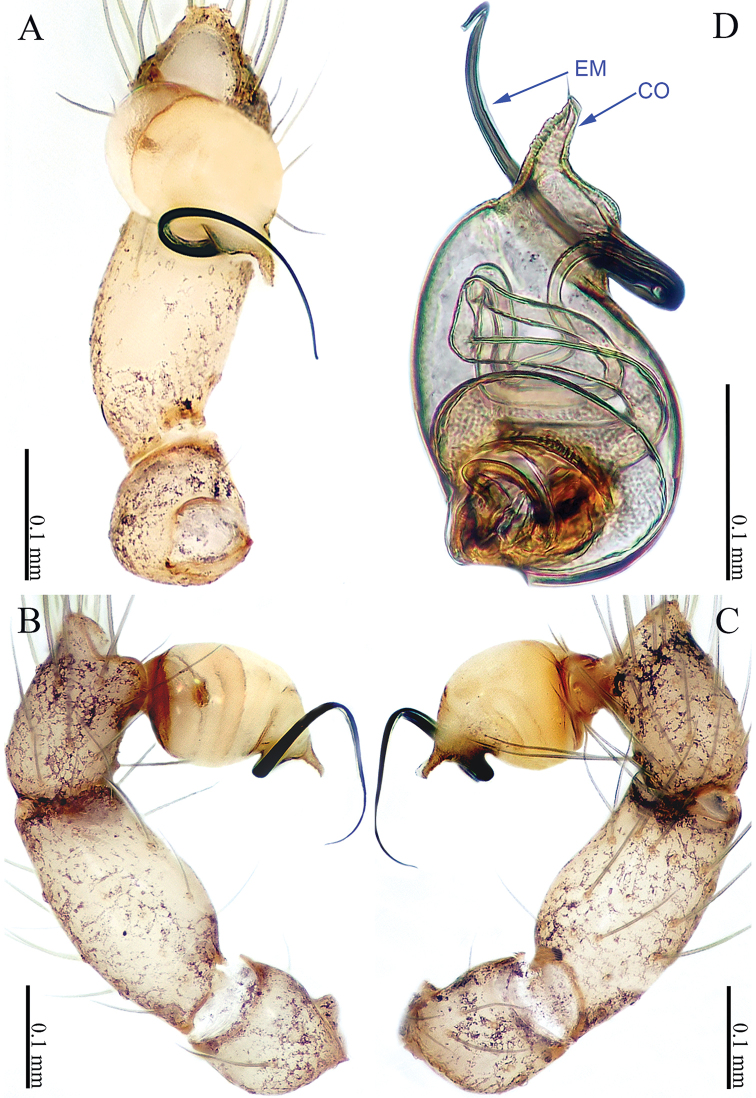
*Sinoderces luohanensis* Li & Li, sp. nov., left pedipalp of male holotype **A** palp, ventral view **B** palp, prolateral view **C** palp, retrolateral view **D** palpal bulb, ventral view. Abbreviations: CO conductor, EM embolus.

**Figure 4.**
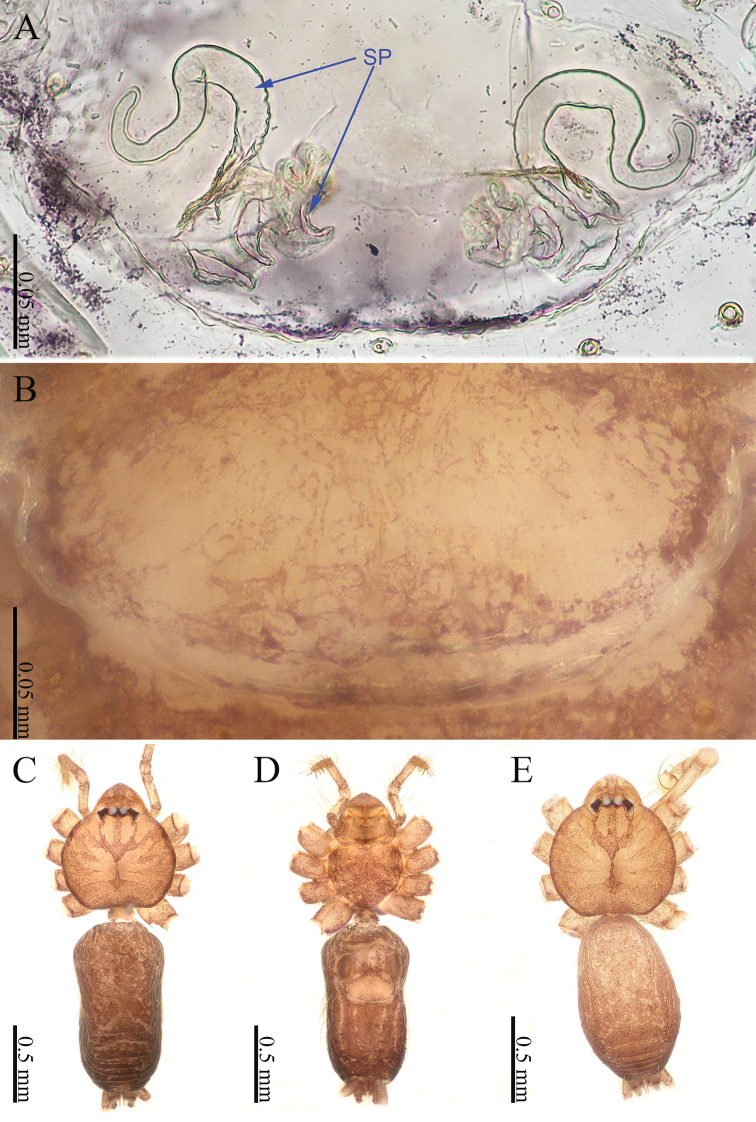
*Sinoderces luohanensis* Li & Li, sp. nov., male holotype and female paratype **A** internal genitalia, dorsal view **B** female epigastric furrow, ventral view **C** Female habitus, dorsal view **D** female habitus, ventral view **E** male habitus, dorsal view. Abbreviation: SP spermathecae.

Figs 3 , 4 , 21 , 22

##### Types.

***Holotype***: ♂, outside of Luohan Cave, Suwei Town, Mu Village, Nanning City, Guangxi Zhuang Autonomous Region, China, 22°32.600'N, 108°03.390'E, 270 m, 9.V. 2015, Chen Z. and Li F. leg. ***Paratypes***: 3♀, same data as holotype.

##### Etymology.

The specific name refers to the name of the cave; adjective.

##### Diagnosis.

*Sinoderces luohanensis* sp. nov. resembles *S. nawanensis* in shape and size (Fig. 4C–E). Males can be distinguished from the latter by the pointed tip of the conductor (vs. blunt tip in *S. nawanensis*) (Fig. 3). Females can be distinguished by the hairless upper part of abdomen (vs. a row of hairs on the same position in *S. nawanensis*) (Fig. 4B). The two pairs of spermathecae have a similar shape, but the terminus of the long pair of spermathecae bends upward in *S. luohanensis* sp. nov. (vs. bending downward in *S. nawanensis*) (Fig. 4A).

##### Description.

**Male** (holotype). Total length 1.66; carapace 0.66 long, 0.71 wide; abdomen 1.00 long, 0.64 wide. Carapace round, brown, with darker brown lateral margins. Carapace with large, brown, fork-shaped pattern in the middle (Fig. 4E). Clypeus brown. Cheliceral promargin with two teeth, followed by a lamina; retromargin with no tooth (Fig. 21B). Labium brown. Sternum brown. Legs brownish yellow, with dark brown joints. Leg measurements: I 8.32 (2.28, 0.25, 2.50, 2.16, 1.13) II 6.43 (1.84, 0.25, 1.88, 1.58, 0.88), III 5.05 (1.44, 0.25, 1.50, 1.25, 0.61), IV 7.26 (2.00, 0.25, 2.21, 1.90, 0.90). Abdomen elongated, wrinkled posteriorly. Spinnerets brown (Fig. 4E).

***Male palp*** (Fig. 3A–D): The overall structure is sickle-shaped. Conductor tip pointed. Embolus curved. Bulb yellow, oval. Conductor and embolus clearly separated, with bases at the distal end of the bulb. Terminal tibia hump with long hairs. Femur and trochanter light yellow with isolated hairs.

**Female.** Color similar to male, and body size slightly larger than males (Fig. 4C–E). Abdomen darker than that in males. Clypeus brown. Carapace round, darker than that of males. Labium and sternum dark brown. Measurements: total length 2.20; carapace 0.64 long, 0.70 wide; abdomen 1.25 long, 0.55 wide. Legs brownish yellow, the joint is dark brown. Leg measurements: I 6.53 (1.75, 0.25, 2.00, 1.63, 0.90), II 5.20 (1.37, 0.25, 1.58, 1.25, 0.75), III 4.31 (1.13, 0.25, 1.25, 1.03, 0.65) IV 6.16 (1.72, 0.25, 1.91, 1.50, 0.78). Abdomen elongated, dorsum with white spots and wrinkles, ventrum with white rings. Epigyne pale yellow. Spinnerets brown.

***Epigyne*** (Fig. 4A, B): Epigynal area lighter in color than other parts of the abdomen. Two pairs of elongated, curved spermathecae. Terminus of the lateral pair of spermathecae bends upward (Fig. 4A).

##### Distribution.

Known only from the type locality (Fig. 22).

##### Natural history.

Collected at a cave entrance at an elevation of 196 m.

#### Sinoderces phathaoensis

Taxon classificationAnimaliaAraneaePsilodercidae

Li & Li sp. nov.

B7665822-9FDE-58E1-8D6F-E746AF79B6D9

http://zoobank.org/A0255BE4-7363-4F92-BD0F-DCCDB72D4561

**Figure 5.**
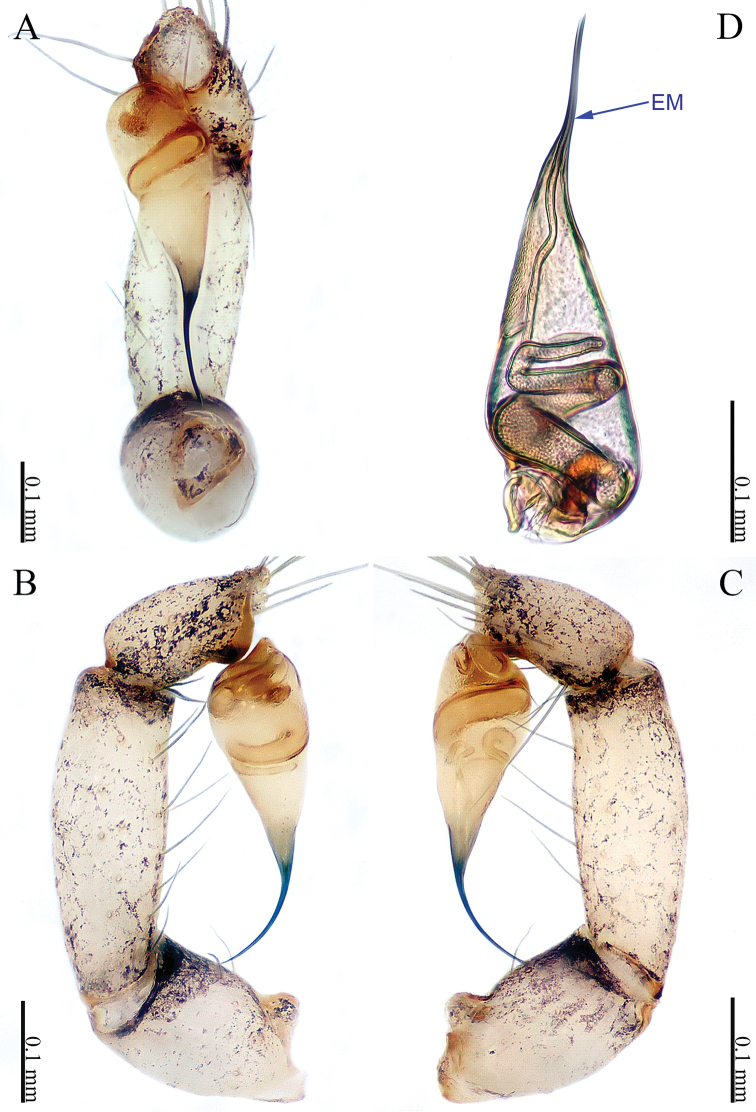
*Sinoderces phathaoensis* Li & Li, sp. nov., left pedipalp of male holotype **A** palp, ventral view **B** palp, prolateral view **C** palp, retrolateral view **D** palpal bulb, ventral view. Abbreviation: EM embolus.

**Figure 6.**
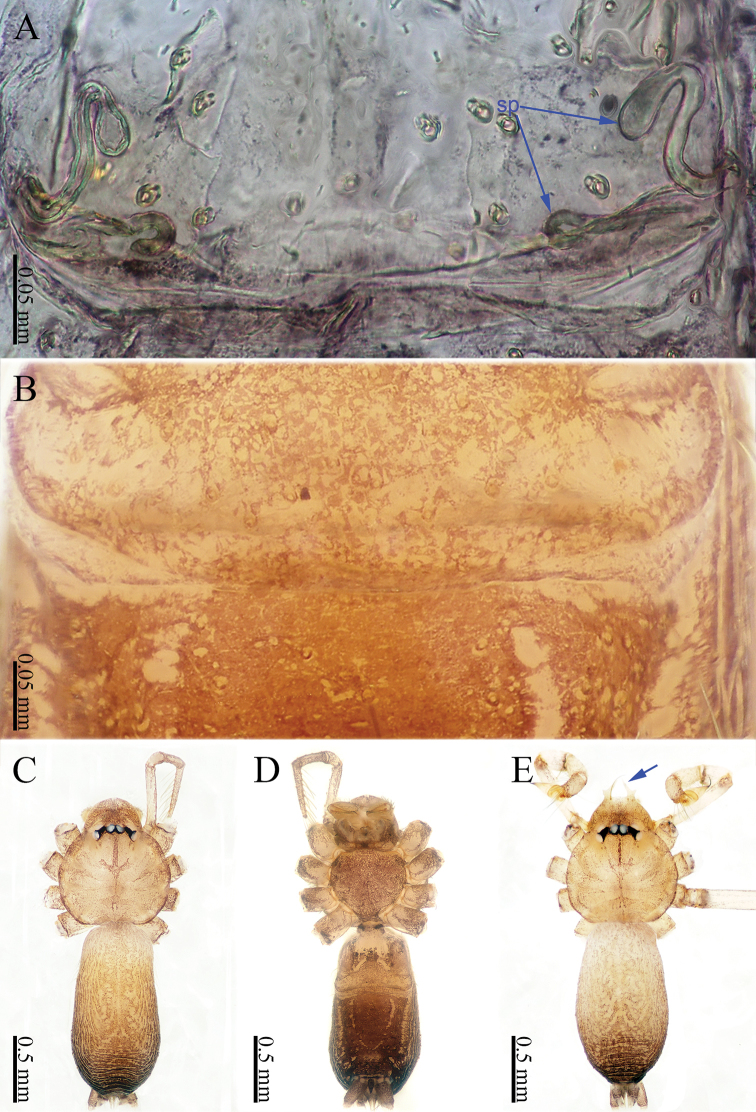
*Sinoderces phathaoensis* Li & Li, sp. nov., male holotype and female paratype **A** internal genitalia, dorsal view **B** female epigastric furrow, ventral view **C** female habitus, dorsal view **D** female habitus, ventral view **E** male habitus, dorsal view (Arrow: apophysis). Abbreviation: SP spermathecae.

Figs 5 , 6 , 21 , 22

##### Types.

***Holotype***: ♂, Pha Thao Cave, Vang Vieng District, 11.95 km north of Viengkieo Village, Vientiane Province, Laos, 19°01.749'N, 102°25.954'E, 290 m, 03.XII.2012, Yao Z. leg. ***Paratypes***: 3♀, same data as holotype

##### Etymology.

The specific name refers to the name of cave; adjective.

##### Diagnosis.

*Sinoderces phathaoensis* sp. nov. resembles *S. kieoensis* sp. nov. in having a similar shaped bulb in males. Males can be distinguished by the curved embolus, almost half as long as the bulb (Fig. 5B, C), in contrast to the straight embolus, less than half the bulb length in *S. kieoensis* sp. nov. (Fig. 18B–D). The bulb of *S. phathaoensis* sp. nov. tapers and narrows more sharply (Fig. 5) than that of *S. kieoensis* sp. nov. (Fig. 18). The male clypeus has two crotched apophyses.

##### Description.

**Male** (holotype). Total length 1.84; carapace 0.64 long, 0.70 wide; abdomen 1.20 long, 0.60 wide. Carapace round, light yellow, with brown lateral margins and a trifurcate mark in the center of the carapace (Fig. 6E). Clypeus slanting and darker brown, medially with one pair of bifurcate apophyses. Labium light yellow. Sternum yellow. The opisthosoma darkens anteriorly to posteriorly (Fig. 6E). Cheliceral promargin with one tooth, connected to a lamina, retromargin with one small tooth (Fig. 21C). Leg measurements: I missing, II missing, III missing, IV missing. Abdomen elongated, wrinkles posteriorly; the color darkens towards the posterior end (Fig. 6E). Spinnerets yellow.

***Male palp*** (Fig. 5A–D): The whole structure is relatively simple. Bulb yellow, conical. Embolus arising distally from the bulb, no conductor. Tibia projected distally. Tibia dark yellow, femur and trochanter light yellow.

**Female** (one of the paratypes). Size and color similar to male (Fig. 6C–E). Total length 1.90; carapace 0.64 long, 0.70 wide; abdomen 1.26 long, 0.60 wide. Carapace brown, with brown lateral margins and a trifurcate mark in the center of carapace (Fig. 6C). Clypeus brown. Endites and labium dark brown. Sternum brown. Legs brownish yellow, with dark brown joints. Leg measurements: I missing, II missing, III 5.58 (1.60, 0.20, 1.70, 1.44, 0.64), IV – (2.20, 0.25, –, –, –). Abdomen wrinkled posterodorsally; the color darkens from anterior to posterior. Ventrum with white rings (Fig. 6E). Spinnerets dark yellow.

***Epigyne*** (Fig. 6A, B): The epigyne is wide and extends to the edge of the abdomen (Fig. 6B). Two pairs of curved spermathecae, the anterior pair of spermathecae resemble a germinating seedling, and the other pair is like a twisted hook (Fig. 6A).

##### Distribution.

Known only from the type locality (Fig. 22).

##### Natural history.

Collected in a cave entrance at an elevation of 290 m.

#### Sinoderces dewaroopensis

Taxon classificationAnimaliaAraneaePsilodercidae

Li & Li sp. nov.

D81C6B52-D934-5580-812B-BEEF4341355C

http://zoobank.org/73BC9150-2CA1-4BAD-A9A8-EFC1D0D3A600

**Figure 7.**
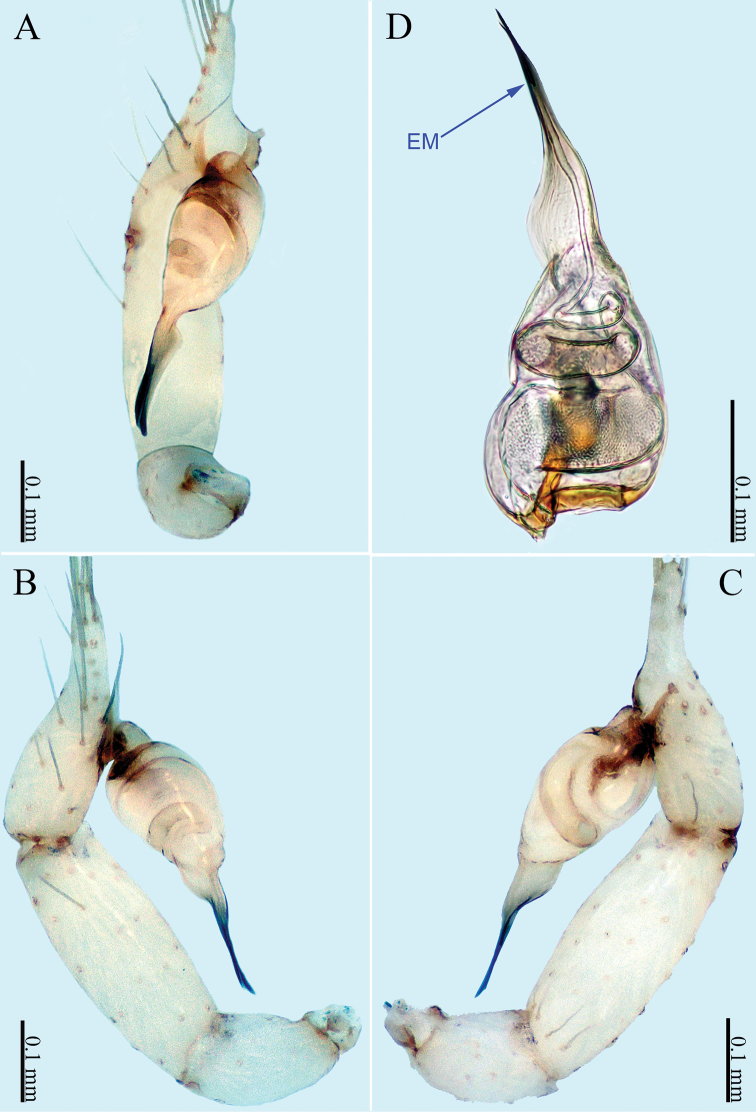
*Sinoderces dewaroopensis* Li & Li, sp. nov., right pedipalp of male holotype **A** palp, ventral view **B** palp, prolateral view **C** palp, retrolateral view **D** palpal bulb, ventral view. Abbreviation: EM embolus.

**Figure 8.**
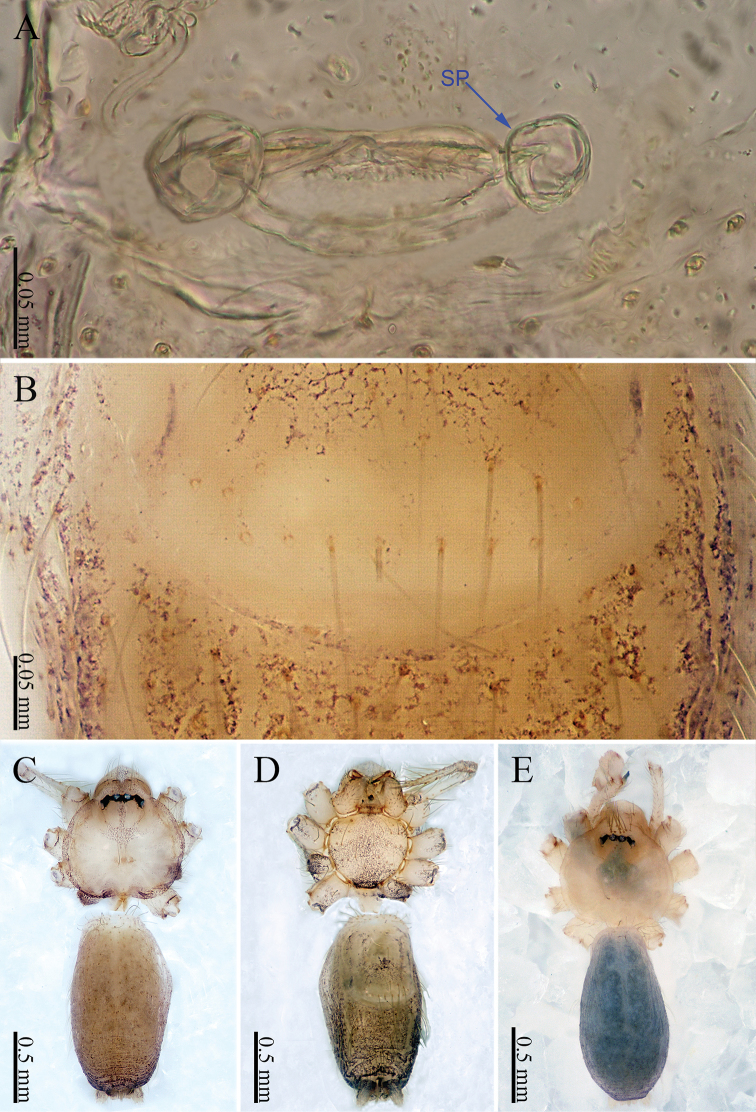
*Sinoderces dewaroopensis* Li & Li, sp. nov., male holotype and female paratype **A** internal genitalia, dorsal view **B** female epigastric furrow, ventral view **C** female habitus, dorsal view **D** female habitus, ventral view **E** male habitus, dorsal view. Abbreviation: SP spermathecae.

Figs 7 , 8 , 21 , 22

##### Types.

***Holotype***: ♂, Dewaroop Cave 1, Pak Chong Distict, Musee Village, Nakhon Ratchasima Province, Thailand, 14°33.708'N, 101°24.064'E, 397 m, 23.X.2014, Zhao H., Li Y. and Chen Z. leg. ***Paratype***: 1♀, same data as holotype.

##### Etymology.

The specific name refers to the name of the cave; adjective.

##### Diagnosis.

*Sinoderces dewaroopensis* sp. nov. can be distinguished from all other known species of the genus by the large apical protrusion on the cymbium (Fig. 7) and a characteristically lamellar embolus. Females can be distinguished by one pair of spherical spermathecae (Fig. 8A)

##### Description.

**Male** (holotype). Total length 1.44; carapace 0.76 long, 1.69 wide; abdomen 1.06 long, 0.54 wide. Carapace round, pale yellow. The overall color is light yellow (Fig. 8E). Cheliceral promargin with one tooth, connected to a lamina; retromargin with one small tooth (Fig. 21D). Clypeus light brown (Fig. 8E). Labium and sternum almost transparent. Legs light yellow, with dark yellow joints. Legs measurements: I 11.03 (3.20, 0.31, 3.28, 3.16, 1.08), II 11.03 (3.08, 0.31, 3.36, 3.20, 1.08), III 11.63 (3.32, 0.31, 3.72, 3.20, 1.08), IV8.07 (2.28, 0.31, 2.44, 2.20, 0.84). Abdomen elongated, with pale yellow (Fig. 8E). Spinnerets pale yellow.

***Male palp*** (Fig. 7A–D): Bulb pale yellow, conical. Embolus arising distally from the bulb, slightly curved; no conductor; tibia with a stout apical protrusion, tipped with many bristles (Fig. 7A). Femur and trochanter light yellow with few hairs.

**Female** (one of the paratypes). Size and color similar to male, but slightly larger and darker (Fig. 8C, D). Total length 1.55; carapace 0.55 long, 0.65 wide; abdomen 1.00 long, 0.55 wide. Carapace round, yellow. Clypeus brown (Fig. 8C). Labium and sternum yellow. Legs light yellow, the joint is dark. Leg measurements: I 9.14 (2.48, 0.25, 2.72, 2.53, 1.16) II 7.06 (1.88, 0.25, 2.05, 1.98, 0.90) III 5.71 (1.53, 0.25, 1.65, 1.50, 0.78), IV 7.81 (2.18, 0.25, 2.33, 2.00, 1.05,). Abdomen elongated, dorsum with black spots and wrinkles, ventrum darkens posteriorly (Fig. 8C). Spinnerets yellow.

***Epigyne*** (Fig. 8A, B): Some bristles on the epigynum (Fig. 8B). It has one pair of global spermathecae (Fig. 8A).

##### Distribution.

Known only from the type locality (Fig. 22).

##### Natural history.

Collected at a cave entrance at an elevation of 397 m.

#### Sinoderces xueae

Taxon classificationAnimaliaAraneaePsilodercidae

Li & Li sp. nov.

367E896C-F713-539A-9A97-0542D49138A4

http://zoobank.org/AC29E243-6C39-45BD-A604-01533D919603

**Figure 9.**
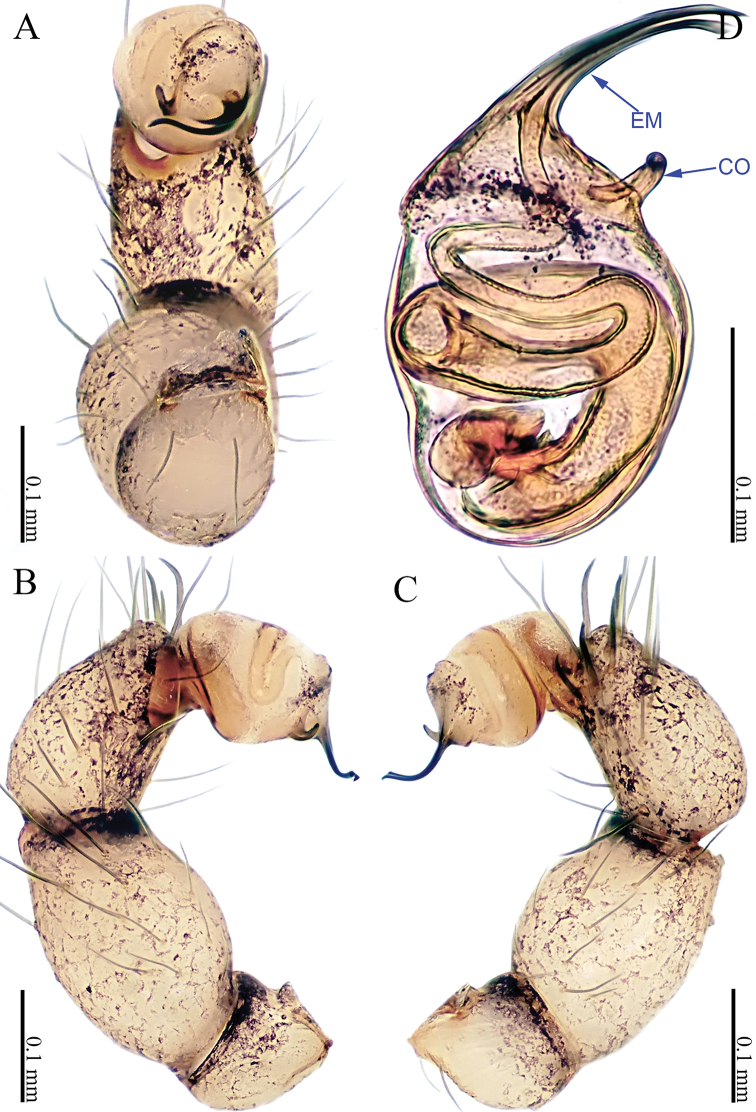
*Sinoderces xueae* Li & Li, sp. nov., male holotype **A** palp, ventral view **B** palp, prolateral view **C** palp, retrolateral view **D** palpal bulb, ventral view Abbreviations: CO conductor, EM embolus.

**Figure 10.**
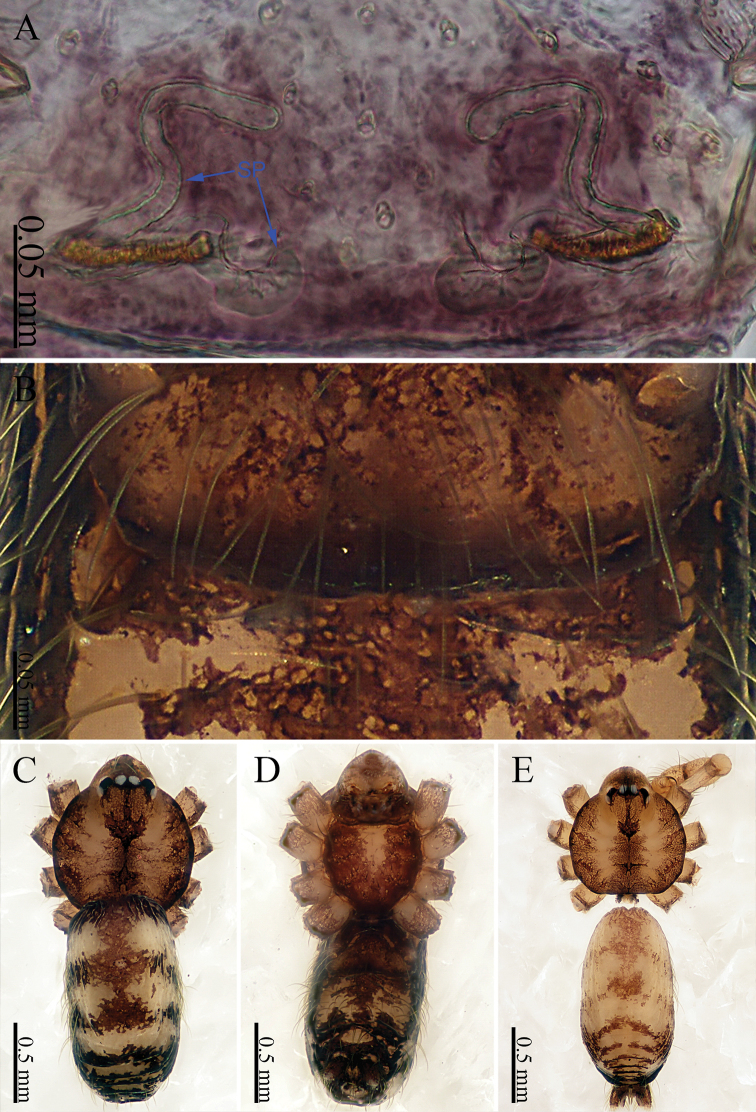
*Sinoderces xueae* Li & Li, sp. nov., male holotype and female paratype **A** internal genitalia, dorsal view **B** female epigastric furrow, ventral view **C** female habitus, dorsal view **D** female habitus, ventral view **E** male habitus, dorsal view. Abbreviation: SP spermathecae.

Figs 9 , 10 , 21 , 22

##### Types.

***Holotype***: ♂, Limu Mountain Town, Hainan Province, China. 19°12.002'N, 109°43.710'E, 591 m, 25.III.2012, Chen Z. leg. ***Paratype***: 1♀, same data as holotype.

##### Etymology.

This name is in honor of Wenjing Xue, a good friend of the first author of the paper who has been helpful to his study and life. The case is feminine and genitive.

##### Diagnosis.

*Sinoderces xueae* sp. nov. resembles *S. taichi* sp. nov. in having a similarly shaped conductor and embolus. However, the males can be distinguished by the following: five spines on the distal part of the palpal cymbium, vs. six in *S. taichi* sp. nov; the slightly sigmoid embolus and short conductor (Fig. 9) vs. a coiled embolus and long conductor in *S. taichi* sp. nov. The margin of the epigyne of *S. xueae* sp. nov. is thin (Fig. 10B) whereas in *S. taichi* sp. nov. thick (Fig. 12B).

##### Description.

**Male** (holotype). Total length 1.94; carapace 0.66 long, 0.72 wide; abdomen 1.28 long, 0.59 wide. Carapace round, brown, with dark brown lateral margins and one wide median brown band, clypeus yellow and chelicerae dark brown (Fig. 10E). Cheliceral promargin with one tooth, followed by a lamina, retromargin with a small tooth (Fig. 21E). Labium brown. Sternum brown (Fig. 10E). Legs yellow, joints darker. Legs measurements: I missing, II 6.26 (1.35, 0.25, 2.13, 1.80, 0.73), III 4.93 (1.40, 0.25, 1.43, 1.25, 0.60), IV 7.26 (2.00, 0.25, 2.25, 1.93, 0.83,). Abdomen elongated, with black lines posteriorly, black anterior lines brownish and expanded medially, and splotches ventrally sp. Spinnerets brown.

***Male palp*** (Fig. 9A–D): The overall color is yellow, bulb dark yellow, ovate; embolus arising retrolaterally and distally from the bulb, slightly sigmoid. Conductor arising prolaterally and proximally from the bulb. Embolus and conductor slightly separated (distance less than a diameter of the bulb). Tibia with five spines distally (Fig. 9B). Femur and trochanter yellow with few hairs.

**Female** (one of the paratypes). Females are darker than males (Fig. 10C, D). Total length 2.08; carapace 0.63 long, 0.63 wide; abdomen 1.45 long, 0.53 wide. Carapace round, brown. Clypeus and chelicerae dark brown (Fig. 10C). Endites dark yellow. Labium and sternum brown. Legs light yellow, joints darker. Leg measurements: I 4.86 (1.20, 0.20, 1.50, 1.24, 0.72), II 3.89 (1.03, 0.20, 1.16, 0.97, 0.53), III 4.95 (1.25, 0.20, 1.44, 1.28, 0.78), IV3.36 (0.91, 0.20, 0.94, 0.78, 0.53). Abdomen elongated, dorsum wrinkled, with black rings, ventrum with large black spots (Fig. 10C, D). Spinnerets black.

***Epigyne*** (Fig. 10A, B): Some bristles present above the epigyne (Fig. 10B). Internal genitalia with two pairs of spermathecae, curved, the anterior pair is longer than the posterior pair (Fig. 10A). The whole entire spermathecal structure is symmetrical.

##### Distribution.

Known only from the type locality (Fig. 22).

##### Natural history.

Collected in pristine forests.

#### Sinoderces taichi

Taxon classificationAnimaliaAraneaePsilodercidae

Li & Li sp. nov.

602D14EE-71F7-557B-B6EC-95D4E29FFAB6

http://zoobank.org/91D5EBDE-5C57-4325-BB99-7D19906C45ED

**Figure 11.**
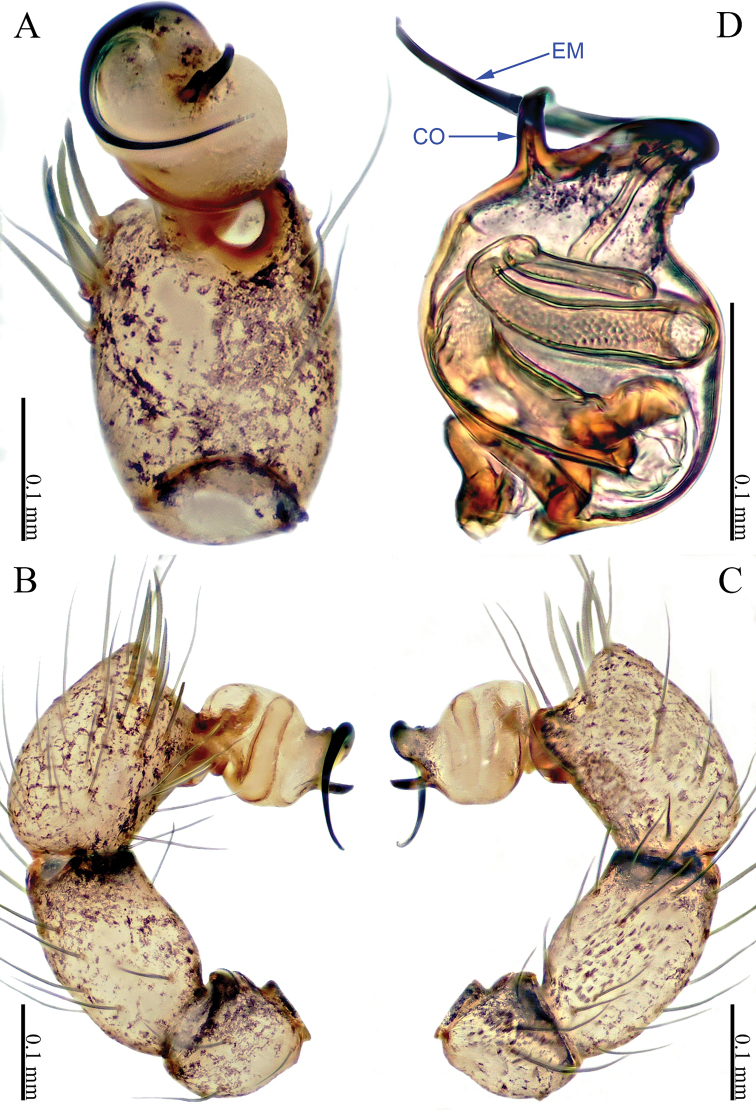
*Sinoderces taichi* Li & Li, sp. nov., male holotype **A** palp, ventral view **B** palp, prolateral view **C** palp, retrolateral view **D** palpal bulb, ventral view Abbreviations: CO conductor, EM embolus.

**Figure 12.**
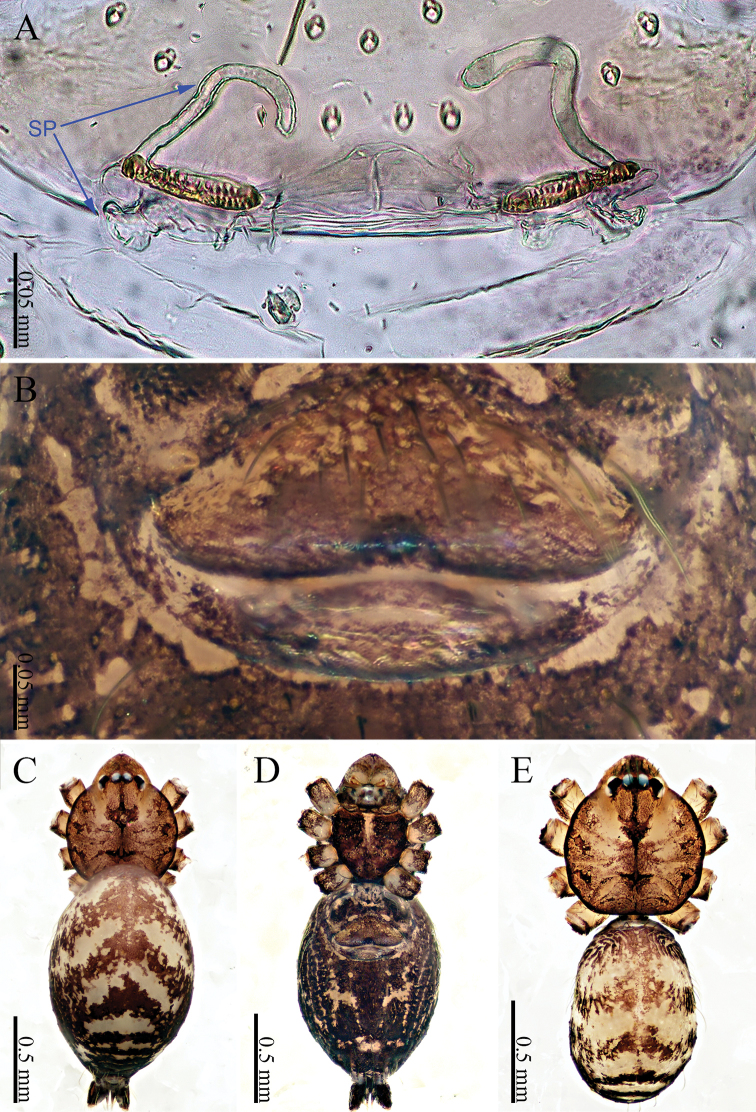
*Sinoderces taichi* Li & Li, sp. nov., male holotype and female paratype **A** internal genitalia, dorsal view **B** female epigastric furrow, ventral view **C** female habitus, dorsal view **D** female habitus, ventral view **E** male habitus, dorsal view. Abbreviation: SP spermathecae.

Figs 11 , 12 , 21 , 22

##### Types.

***Holotype***: ♂, Lingshui Town, Hainan Province, China. 18°43.777'N, 109°51.740'E, 03.IV.2012, Chen Z. leg. ***Paratypes***: 2♀, same data as holotype.

##### Etymology.

In ventral view (Fig. 11A), the embolus and conductor resemble the yin-yang symbol representing the philosophy behind the Chinese martial art of Taichi; noun in apposition.

##### Diagnosis.

*Sinoderces taichi* sp. nov. resembles *S. xueae* sp. nov. in having a similarly shaped conductor and embolus. However, the males can be distinguished by the following: six spines on the distal part of the palpal cymbium, as against vs. five in *S. xueae* sp. nov.; a coiled embolus and long conductor. The edge margin of the epigyne is very thick (Fig. 12B).

##### Description.

Male (holotype). Total length 1.56; carapace 0.70 long, 0.67 wide; abdomen 0.86 long, 0.64 wide. Carapace round, brown, with dark brown margins and a narrow, brown median line behind ocular area (Fig. 12E). Clypeus dark yellow and chelicerae dark yellow. Cheliceral promargin with one tooth, followed by a lamina, retromargin with one small tooth (Fig. 21F). Endites dark yellow. Labium brown. Sternum dark yellow. Legs brown, joints darker. Leg measurements: I 8.72 (2.44, 0.20, 2.88, 2.40, 0.80), II missing, III 4.98 (1.50, 0.20, 1.40, 1.20, 0.68), IV 7.32 (2.00, 0.20, 2.28, 1.84, 1.00). Abdomen wrinkled posterodorsally, with wide brown markings (Fig. 12E). Spinnerets black.

***Male palp*** (Fig. 11B): Short and thick. Bulb dark yellow, ovate. Embolus arising prolaterally and proximally from the bulb, coiled (Fig. 11). Conductor arising retrolaterally and distally from the bulb. Embolus and conductor slightly separated (distance less than diameter of bulb). Distal part of the tibia with a row of six spines. Femur and trochanter dark yellow with few hairs.

**Female** (one of the paratypes). Size and color similar to the male but slightly larger and darker (Fig. 12C, D). Total length 2.125; carapace 0.63 long, 0.63 wide; abdomen 1.50 long, 0.88 wide. Carapace round and brown. Clypeus brown. Chelicerae dark yellow. Endites dark yellow. Labium and sternum dark brown. Legs brown, joints dark. Leg measurements: I 5.28 (1.32, 0.20, 1.64, 1.30, 0.82), II missing, III 3.48 (0.90, 0.20, 0.90, 0.86, 0.62), IV4.92 (1.22, 0.20, 1.52, 1.24, 0.74). Abdomen elongated, dorsum with regular black rings and wrinkles, ventrum with yellow spots (Fig. 12C, D). Spinnerets black.

***Epigyne*** (Fig. 12A, B): Some bristles present above the epigyne (Fig. 12B). The anterior pair is a symmetric c shape. The anterior pair are tubular structures. The posterior pairs are membranous (Fig. 12A).

##### Distribution.

Known only from the type locality (Fig. 22).

##### Natural history.

Collected in pristine forest within a protected zone.

#### Sinoderces wenshanensis

Taxon classificationAnimaliaAraneaePsilodercidae

Li & Li sp. nov.

348192A8-6B82-5A86-841E-5B83D11220EC

http://zoobank.org/34E03784-9051-4EF7-96E2-E36BD6ADA28C

**Figure 13.**
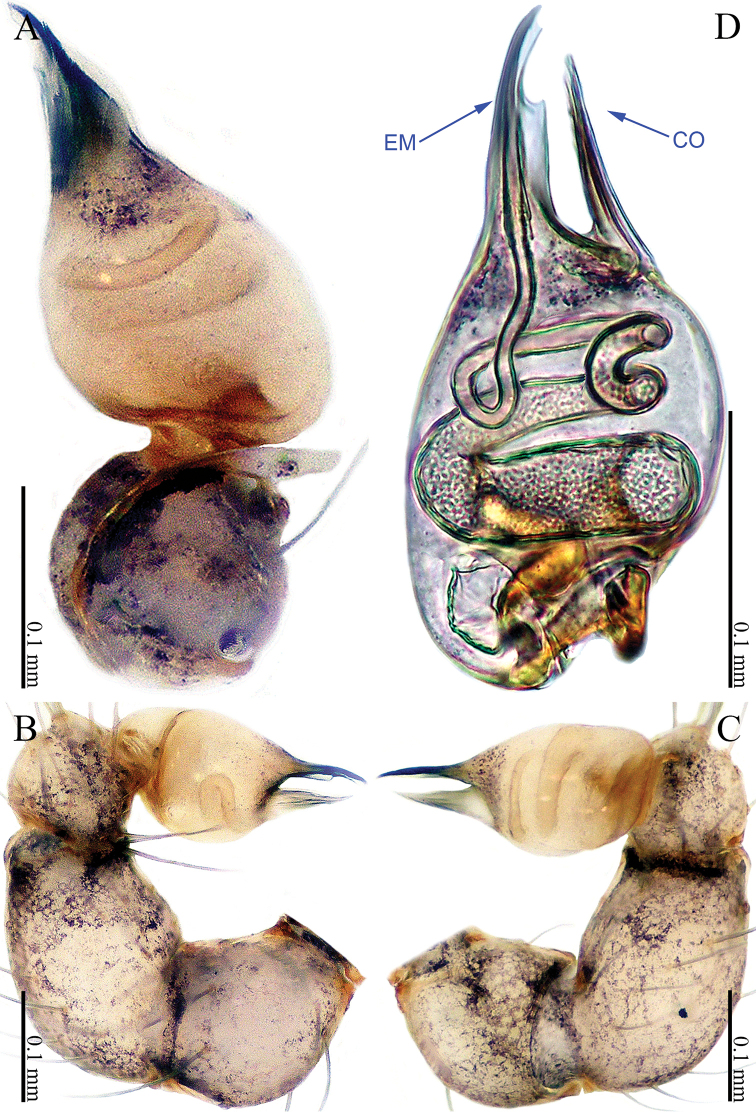
*Sinoderces wenshanensis* Li & Li, sp. nov., male holotype **A** palp, ventral view **B** palp, prolateral view **C** palp, retrolateral view **D** palpal bulb, ventral view Abbreviations: CO conductor, EM embolus.

**Figure 14.**
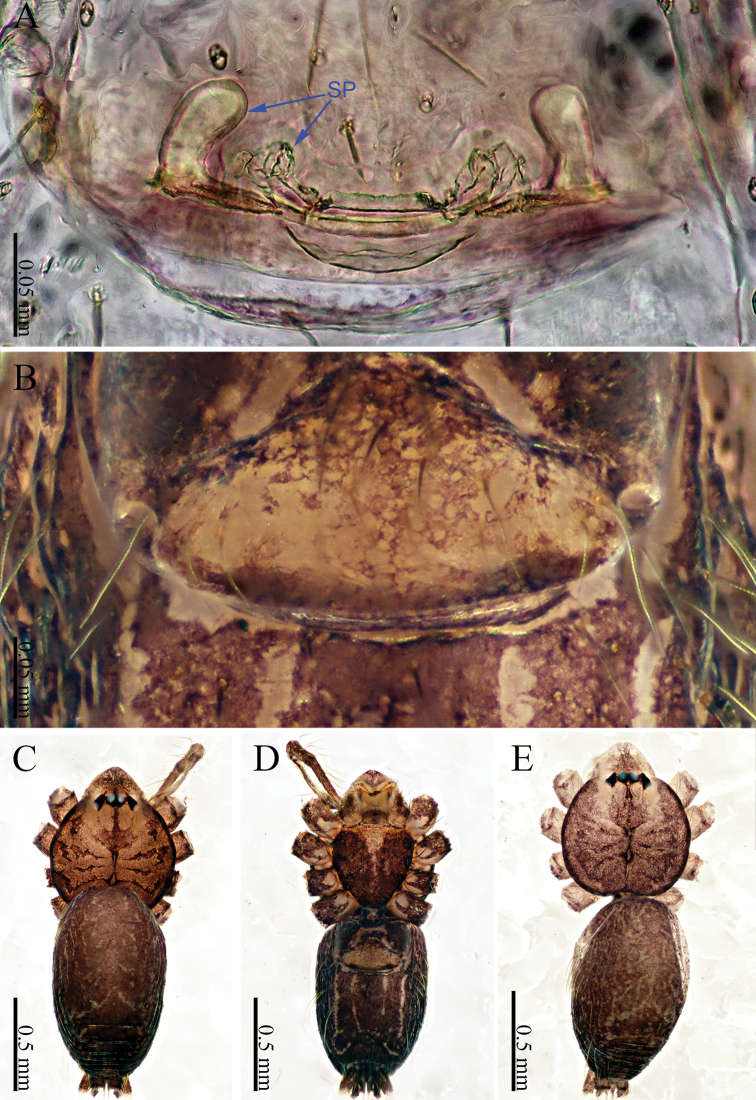
*Sinoderces wenshanensis* Li & Li, sp. nov., male holotype and female paratype **A** internal genitalia, dorsal view **B** female epigastric furrow, ventral view **C** female habitus, dorsal view **D** female habitus, ventral view **E** male habitus, dorsal view. Abbreviation: SP spermathecae.

Figs 13 , 14 , 21 , 22

##### Types.

***Holotype***: ♂, Radio and television station hill, Xiqiao County, Wenshan, Yunnan Province, China, 23°25.980'N, 104°40.392'E, 1556 m, 17.V.2015, Li F. and Chen Z. leg. ***Paratypes***: 2♀, same data as holotype.

##### Etymology.

The specific name refers to the name of the hill at the type locality; adjective.

##### Diagnosis.

*Sinoderces wenshanensis* sp. nov. can be distinguished from all other known species by the nearly parallel conductor and embolus (Fig. 13D). The conductor is membranous (Fig. 13B–D). Palp nearly light yellow. Entrance duct is visible even before soaking them in lactic acid. Epigyne resembles a fan. Females have two pairs of spermathecae, with the inner pair together resembling the smile of a smiley face and the outer pair protruding anteriorly (Fig. 14A, B)

##### Description.

**Male** (holotype). Total length 1.72; carapace 0.62 long, 0.70 wide; abdomen 1.10 long, 0.60 wide. Carapace round, dark brown, with dark brown margins (Fig. 14E). Clypeus light brown. Chelicerae light yellow. Cheliceral promargin with one tooth, followed by a lamina; retromargin with one small tooth (Fig. 21G). Endites yellow. Labium light brown. Sternum dark brown. Legs brown, joints dark. Leg measurements: I missing, II missing, III missing, IV 7.05 (1.87, 0.25, 2.25, 1.75, 0.93). The abdomen is slightly distorted from preservation (Fig. 14E). Opisthosoma brown. Spinnerets brown.

***Male palp*** (Fig. 13): Bulb light, ovate, Conductor almost parallel to the palpal axis. Embolus arising terminally from the bulb, straight and needle-like. Conductor arising close to embolus, membranous. Embolus and conductor completely separated. Tibia, femur and trochanter light yellow with hairs.

**Female** (paratype). Similar to male in color and general features (Fig. 14C, D). Sternum heart-shaped. Total length 1.72; carapace 0.63 long, 0.70 wide; abdomen 1.09 long, 0.61 wide. Carapace round and brown. Clypeus brown and chelicerae yellow. Endites dark yellow. Labium and sternum brown. Legs brown, joints dark. Leg measurements: I 6.78 (1.75, 0.31, 2.00, 1.72, 1.00), II 5.15 (1.31,0.31,1.56,1.25,0.72), III 4.13 (1.09, 0.31, 1.16, 0.94, 0.63), IV 6.06 (1.56, 0.31, 1.91, 1.47, 0.81). Abdomen elongated, with yellow pinstripe dorsally and ventrally.

***Epigyne*** (Fig. 14A, B): fan-like, brown, with sparse hairs. Other features described under Diagnosis (Fig. 14A, B).

##### Distribution.

Known only from the type locality (Fig. 22).

##### Natural history.

Collected from leaf litter at an elevation of 1556 m.

#### Sinoderces aiensis

Taxon classificationAnimaliaAraneaePsilodercidae

Li & Li sp. nov.

407A9006-ED4D-5F55-A94B-0745B7E74BD2

http://zoobank.org/7DA0FEFC-FB00-457A-B1C4-757D6199B78E

**Figure 15.**
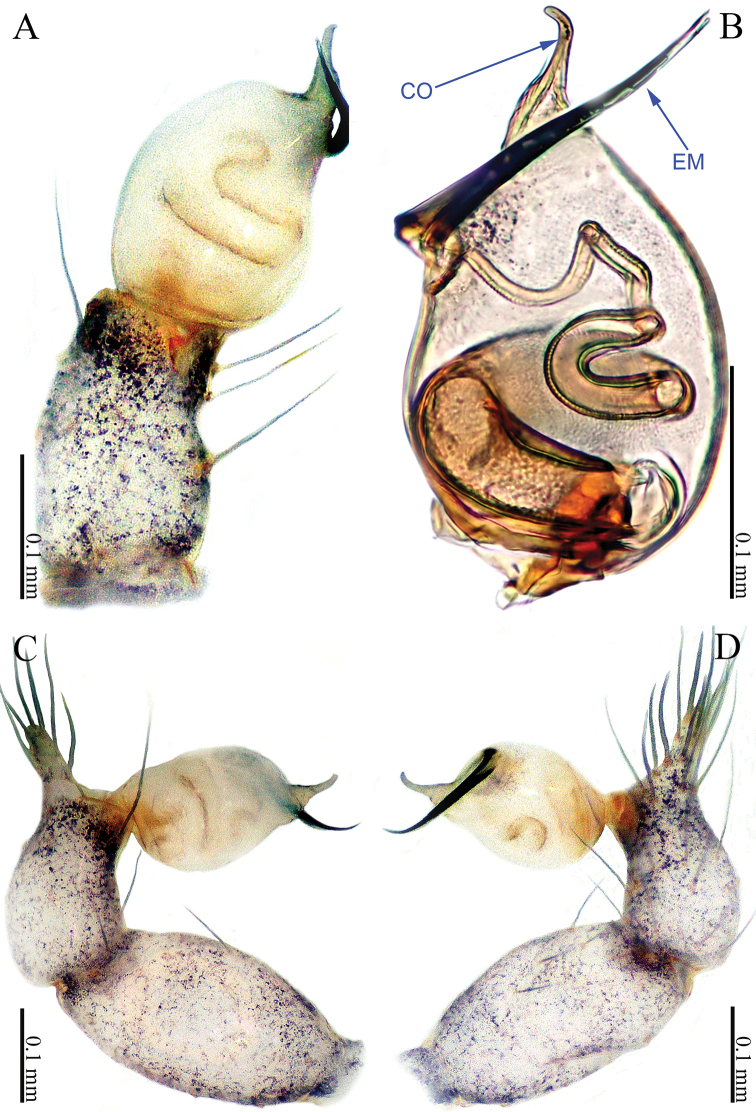
*Sinoderces aiensis* Li & Li, sp. nov., male holotype **A** palp, ventral view **B** palp, prolateral view **C** palp, retrolateral view **D** palpal bulb, ventral view. Abbreviations: CO conductor, EM embolus.

**Figure 16.**
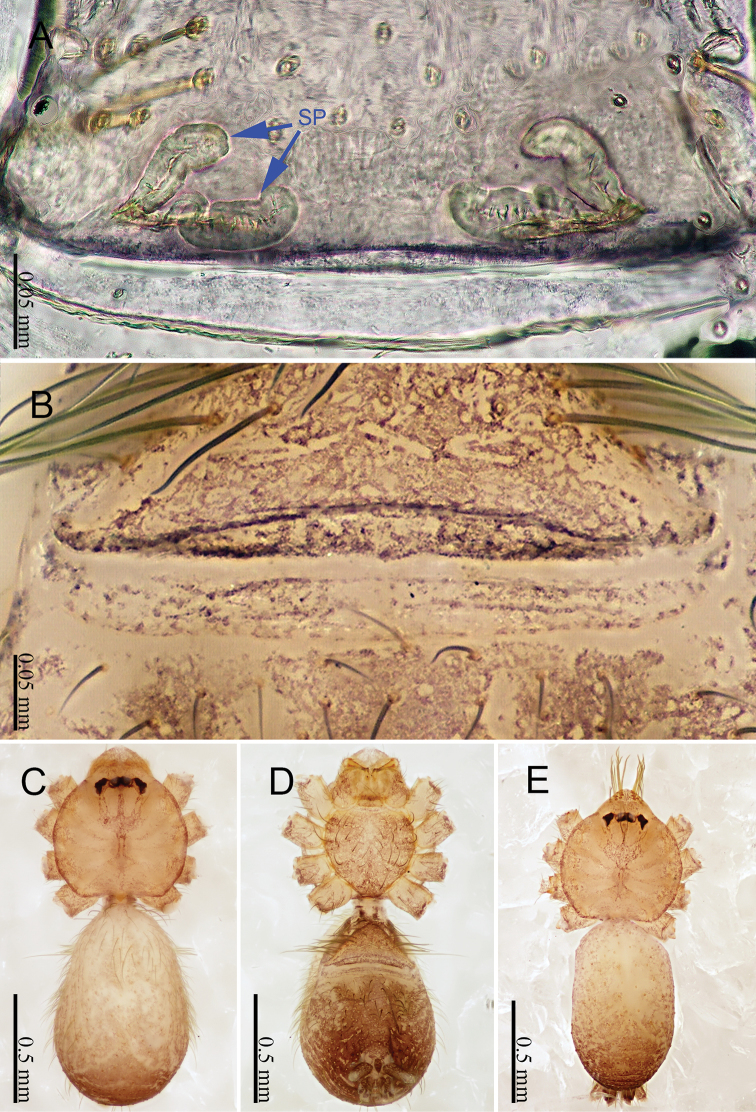
*Sinoderces aiensis* Li & Li, sp. nov., male holotype and female paratype **A** internal genitalia, dorsal view **B** female epigastric furrow, ventral view **C** female habitus, dorsal view **D** female habitus, ventral view **E** male habitus, dorsal view. Abbreviation: SP spermathecae.

Figs 15 , 16 , 21 , 22

##### Types.

***Holotype***: ♂, Ai Cave, Baoyou Village, Qicha Town, Changjiang li autonomous County, Hainan Province, China. 19°6.068'N, 109°1.200'E, 125 m, 18.XII. 2014, Zhao Z. and Shao L. leg. ***Paratypes***: 1♂2♀, same data as holotype.

##### Etymology.

The specific name refers to the name of the cave; adjective.

##### Diagnosis.

*Sinoderces aiensis* sp. nov. can be distinguished from all other known species by the light-colored bulb, a curved conductor with a slightly curved tip (Fig. 15) and an embolus shaped like the Nike swoosh logo (Fig. 15B, C). There is a finger-like projection at the junction of the tarsus and the tibia. Females may be recognized by the antler-like spermathecae (Fig. 16A).

##### Description.

**Male** (holotype). Total length 1.31; carapace 0.54 long, 0.50 wide; abdomen 0.77 long, 0.43 wide. Carapace round, light yellow, with darker yellow margins and a narrow, dark yellow median line behind ocular area (Fig. 16E). Clypeus and chelicerae yellow. Cheliceral promargin with one tooth, followed by a lamina, retromargin with one small tooth (Fig. 21H). Endites and labium dark yellow. Sternum yellow. Legs light yellow. Leg measurements: I missing, II missing, III missing, IV 4.14 (1.10, 0.20, 1.20, 1.20, 0.44). Abdomen elongated, gradually darkens from anterior to posterior (Fig. 16E). Spinnerets dark yellow.

***Male palp*** (Fig. 15): The palp is almost light yellow. Bulb light, ovate; conductor with a slightly curved tip and the embolus like the Nike swoosh logo. Tibia with a stout apical protrusion, the protrusion with many bristles (Fig. 15B). Oval femur with hairs.

**Female** (one of the paratypes). Similar to male in light color and general features (Fig. 16C, D) but bigger than males. Total length 1.48; carapace 0.53 long, 0.63 wide; abdomen 0.95 long, 0.63 wide. Carapace yellow. Clypeus and chelicerae dark yellow. Endites and labium dark yellow. Sternum yellow. Legs light yellow. Leg measurements: I 5.36 (1.41, 0.16, 1.72, 1.38, 0.69), II missing, III missing, IV 5.03 (1.31,0.16, 1.59, 1.31, 0.66). Abdomen elongated, gradually darkens from front to back (Fig. 16 C, D). Spinnerets dark yellow.

***Epigyne*** (Fig. 16A, B): Dark yellow, with two hair tufts (16 B). Two pairs of spermathecae that resemble antlers. (Fig. 16A).

##### Distribution.

Known only from the type locality (Fig. 22).

##### Natural history.

Collected from a cave at an elevation of 125 m.

#### Sinoderces saraburiensis

Taxon classificationAnimaliaAraneaePsilodercidae

Li & Li sp. nov.

10E048EC-F9DC-5BA4-85F5-55CD07FB5080

http://zoobank.org/35A1C550-4CCC-4198-8FA0-7864BA50C55F

**Figure 17.**
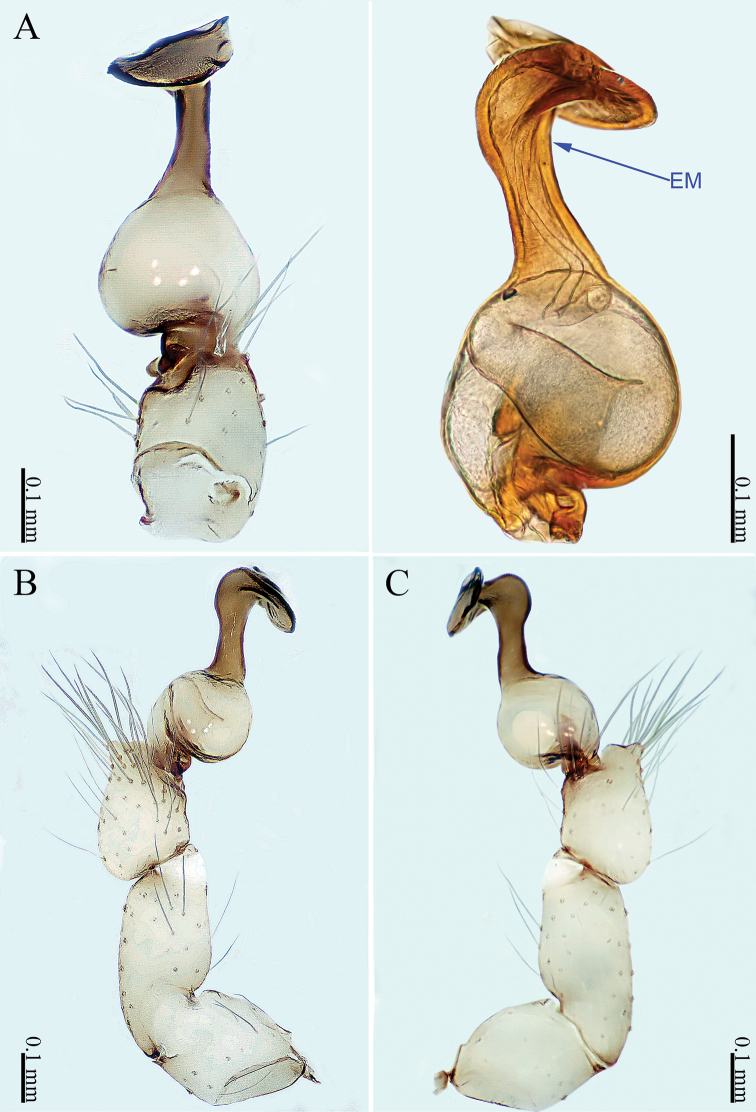
*Sinoderces saraburiensis* Li & Li, sp. nov., male holotype **A** palp, ventral view **B** palp, prolateral view **C** palp, retrolateral view **D** palpal bulb, ventral view Abbreviation: EM embolus.

**Figure 18.**
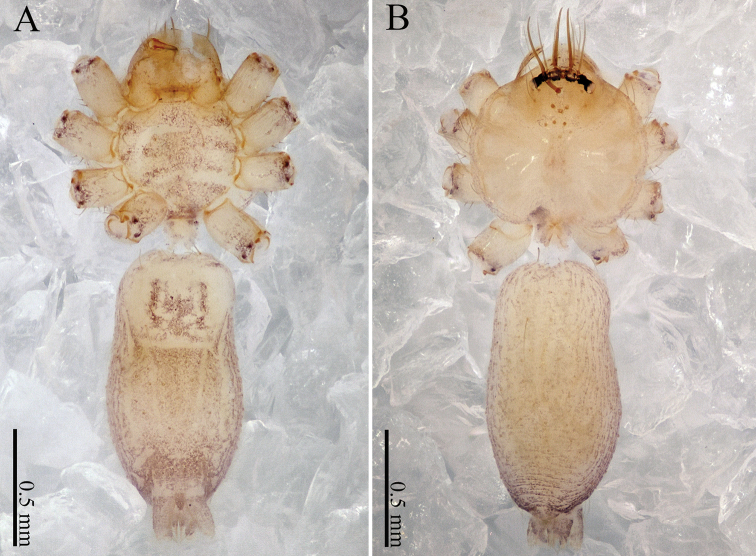
*Sinoderces saraburiensis* Li & Li, sp. nov., male holotype **A** male habitus, ventral view **B** male habitus, dorsal view.

Figs 17 , 18 , 21 , 22

##### Types.

***Holotype***: ♂, Tham Bo Pla Cave, Kaeng Koi District, Song Khon Village, Saraburi Province, Thailand, 14°39.625'N, 100°58.115'E, 73 m, 20. X. 2014, Zhao H., Li Y. and Chen Z. leg.

##### Etymology.

The specific name is derived from the type locality; adjective.

##### Diagnosis.

*Sinoderces saraburiensis* sp. nov. can be distinguished from all other known species by the swan-like shape of the palp (Fig. 17). Embolus heavily sclerotized. Palpal tibia with a slight bump. No conductor.

##### Description.

**Male** (holotype). Total length 1.82; carapace 0.65 long, 0.72 wide; abdomen 1.18 long, 0.56 wide. Carapace round, light yellow, with dark lateral margins. Anterior margin of cephalic region distinctly elevated. Clypeus yellow. Chelicerae light yellow. Cheliceral promargin with one tooth, connected to a lamina; retromargin toothless (Fig. 21I). Endites dark yellow. Labium yellow. Sternum dark yellow. Legs light yellow. Leg measurements: I 13.61 (3.75, 0.31, 4.05, 4.15, 1.35), II 9.49 (2.50, 0.31, 2.81, 2.78, 1.09,). III 6.75 (1.90, 0.25, 2.20, 1.80, 0.60), IV 9.87 (2.72, 0.25, 3.00, 2.81, 1.09). The color of the abdomen gradually darkens posteriorly.

***Male palp*** (Fig. 17): Bulb pale yellow, ovate. Embolus shaped like a swan; the top of tarsus darker than the rest. Embolus is grossly extended and twisted apically. No conductor. Tibia with a slight bump densely covered by bristles. Femur with sparse hairs.

##### Distribution.

Known only from the type locality (Fig. 22).

##### Natural history.

Collected in a cave at an elevation of 73 m.

#### Sinoderces kieoensis

Taxon classificationAnimaliaAraneaePsilodercidae

Li & Li sp. nov.

9DEEB6F3-DCDA-5A62-BB62-0466B9BEBE18

http://zoobank.org/D54B881A-0256-4A59-A7BF-0B07E0BC9F27

**Figure 19.**
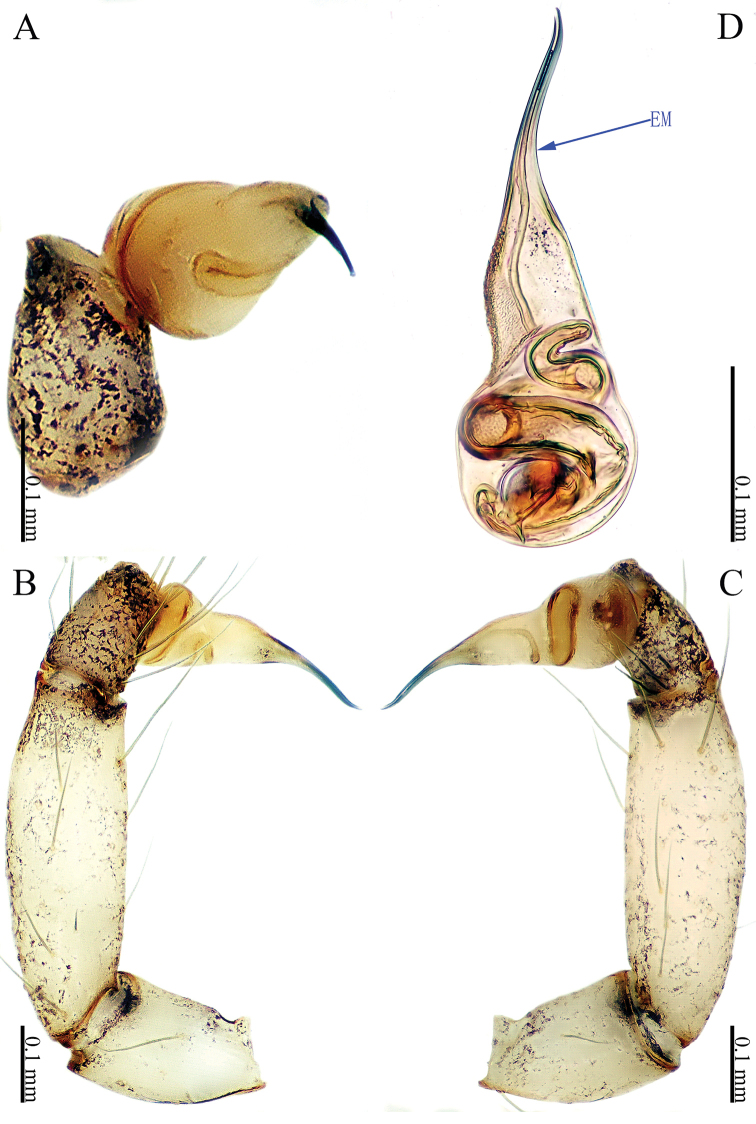
*Sinoderces kieoensis* Li & Li, sp. nov., male holotype **A** palp, ventral view **B** palp, prolateral view **C** palp, retrolateral view **D** palpal bulb, ventral view Abbreviation: EM embolus.

**Figure 20.**
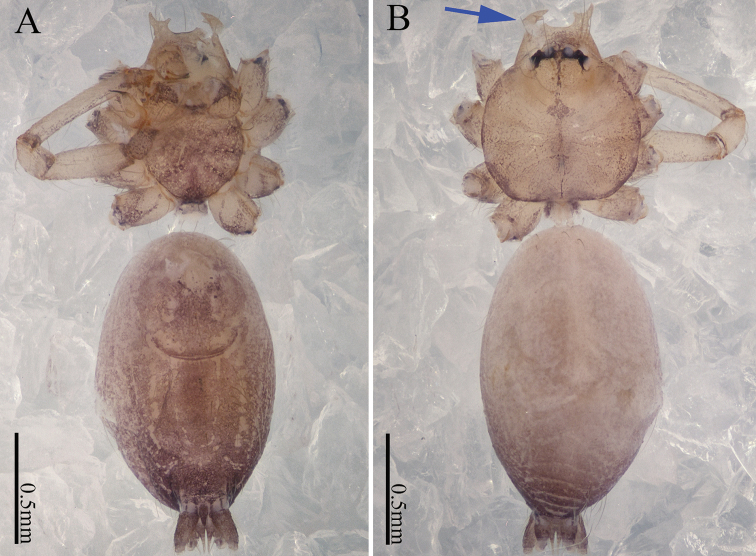
*Sinoderces kieoensis* Li & Li, sp. nov., male holotype **A** male habitus, ventral view **B** male habitus, dorsal view (Arrow: apophysis).

**Figure 21.**
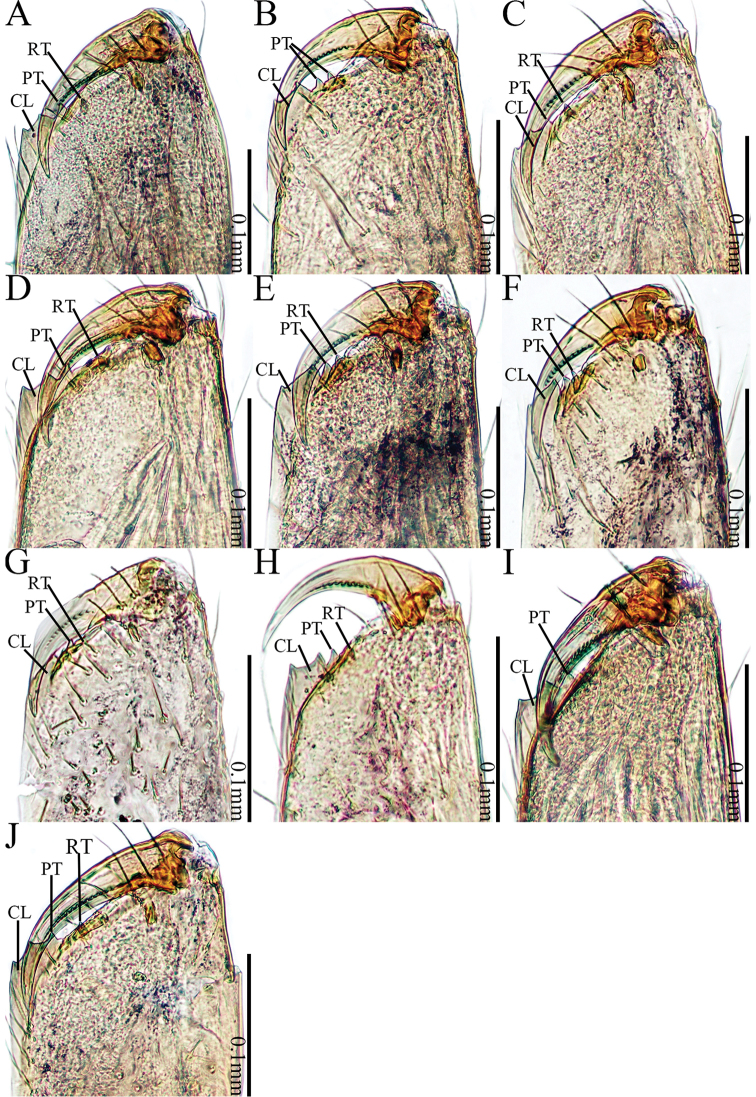
Cheliceral retromargin **A***Sinoderces khanensis* sp. nov. **B***S. luohanensis* sp. nov. **C***S. phathaoensis* sp. nov. **D***S. dewaroopensis* sp. nov. **E***S. xueae* sp. nov. **F***S. taichi* sp. nov. **G***S. wenshanensis* sp. nov. **H***S. aiensis* sp. nov. **I***S. saraburiensis* sp. nov. **J***S. kieoensis* sp. nov. Abbreviations: RT retromargin teeth, PT promarginal teeth, CL cheliceral laminal.

**Figure 22.**
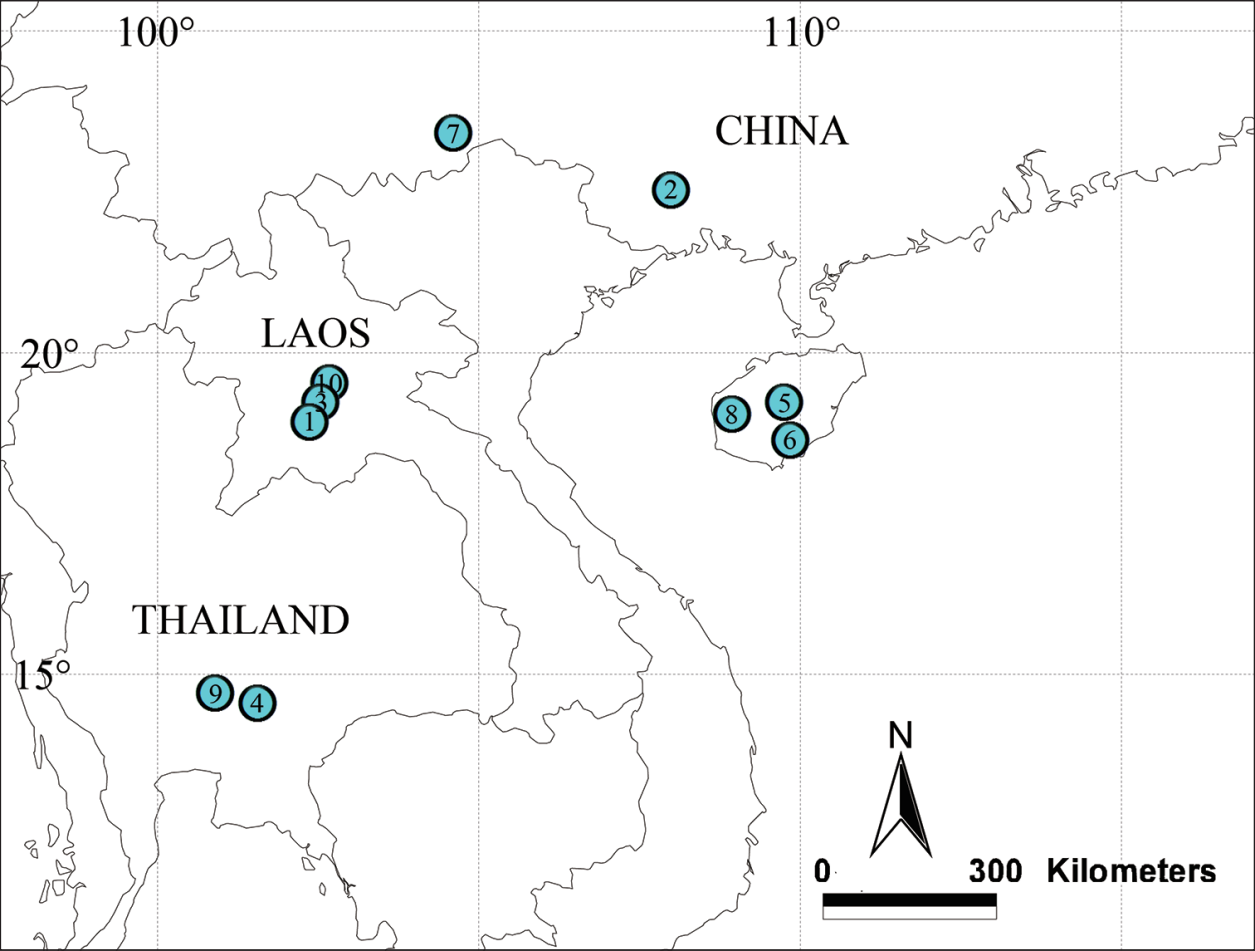
Distribution of ten new *Sinoderces* in China, Laos and Thailand **1***Sinoderces khanensis* sp. nov. **2***S. luohanensis* sp. nov. **3***S. phathaoensis* sp. nov. **4***S. dewaroopensis* sp. nov. **5***S. xueae* sp. nov. **6***S. taichi* sp. nov. **7***S. wenshanensis* sp. nov. **8***S. aiensis* sp. nov. **9***S. saraburiensis* sp. nov. **10***S. kieoensis* sp. nov.

Figs 19 , 20 , 21 , 22

##### Types.

***Holotype***: ♂, Kieo Cave, Vang Vieng District, 10.37 km north of Viengkieo Village, Vientiane Province, Laos, 19°00.880'N, 102°25.902'E, 286 m, 2.XII.2012, Yao Z. leg.

##### Etymology.

The specific name refers to the name of the cave at the type locality; adjective.

##### Diagnosis.

*Sinoderces kieoensis* sp. nov. resembles *S. phathaoensis* sp. nov. in having a similar shaped bulb in males. Males can be distinguished from by the straight embolus, with less than half the bulb length in *S. kieoensis* sp. nov. (Fig. 18B–D), in contrast with to the curved, embolus almost half as long as the bulb (Fig. 5 B, C). The bulb of *S. kieoensis* sp. nov. (Fig. 18) is thicker and more blunt than that of *S. pathaoensis* sp. nov. (Fig. 5).

##### Description.

**Male** (holotype). Total length 2.28; carapace 0.72 long, 0.78 wide; abdomen 1.56 long, 0.63 wide. Carapace round, yellow, with brown lateral margins. The brown line is close to the center with a circular brown spot. Clypeus brown, medially with one pair of bifurcate apophyses. Chelicerae dark yellow. Cheliceral promargin with one tooth, connected to a lamina, retromargin with one small tooth (Fig. 21J). Endites brown. Labium brown. Sternum dark yellow. Leg measurements: I missing, II missing, III missing, IV missing. Abdomen elongated; dorsum dark brown, with yellow stripe; ventrum dark yellow; the color of the abdomen gradually darkens from anterior to posterior.

***Male palp*** (Fig. 18): Bulb yellow, conical. The center of the bulb with slight constriction. Embolus arising distally from bulb, straight. No conductor. Tibia dark yellow. Femur and trochanter light yellow.

##### Distribution.

Known only from the type locality (Fig. 22).

##### Natural history.

Collected in a cave at an elevation of 286 m.

## Supplementary Material

XML Treatment for Sinoderces

XML Treatment for Sinoderces khanensis

XML Treatment for Sinoderces luohanensis

XML Treatment for Sinoderces phathaoensis

XML Treatment for Sinoderces dewaroopensis

XML Treatment for Sinoderces xueae

XML Treatment for Sinoderces taichi

XML Treatment for Sinoderces wenshanensis

XML Treatment for Sinoderces aiensis

XML Treatment for Sinoderces saraburiensis

XML Treatment for Sinoderces kieoensis
